# A two-dimensional *π–d* conjugated coordination polymer with extremely high electrical conductivity and ambipolar transport behaviour

**DOI:** 10.1038/ncomms8408

**Published:** 2015-06-15

**Authors:** Xing Huang, Peng Sheng, Zeyi Tu, Fengjiao Zhang, Junhua Wang, Hua Geng, Ye Zou, Chong-an Di, Yuanping Yi, Yimeng Sun, Wei Xu, Daoben Zhu

**Affiliations:** 1 Beijing National Laboratory for Molecular Sciences, Key Laboratory of Organic Solids, Institute of Chemistry, Chinese Academy of Sciences, Beijing 100190, China; 2 The Daniel Chee Tsui Laboratory, Institute of Physics, Chinese Academy of Sciences, Beijing 100190, China

## Abstract

Currently, studies on organic two-dimensional (2D) materials with special optic-electronic properties are attracting great research interest. However, 2D organic systems possessing promising electrical transport properties are still rare. Here a highly crystalline thin film of a copper coordination polymer, Cu-BHT (BHT=benzenehexathiol), is prepared via a liquid–liquid interface reaction between BHT/dichloromethane and copper(II) nitrate/H_2_O. The morphology and structure characterization reveal that this film is piled up by nanosheets of 2D lattice of [Cu_3_(C_6_S_6_)]_*n*_, which is further verified by quantum simulation. Four-probe measurements show that the room temperature conductivity of this material can reach up to 1,580 S cm^−1^, which is the highest value ever reported for coordination polymers. Meanwhile, it displays ambipolar charge transport behaviour and extremely high electron and hole mobilities (99 cm^2 ^V^−1 ^s^−1^ for holes and 116 cm^2 ^V^−1 ^s^−1^ for electrons) under field-effect modulation.

## Abstract (web summary)

Conjugated 2D materials are able to marry the properties of transparency and conductivity for implementation in a wide range of devices. Here, Huang and *et al.* adopt this design principal in synthesizing a copper bis(dithiolene) coordination polymer which exhibits remarkable electronic performances.

## Graphical abstract

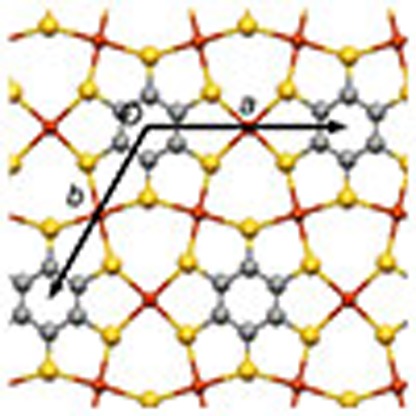

Two-dimensional (2D) materials, such as graphene^1^,^2^,^3^,^4^,^5^, graphdiyne^6^,^7^,^8^, hexagonal boron nitride^9^,^10^ and nanosheets of metal oxides^11^, metal sulfides^12^,^13^,^14^,^15^,^16^, metal hydroxides^17^ and silicon^18^,^19^, have received great research interests. Owing to their unique properties resulting from 2D nature, they can be utilized in spin-based^20^ and photoelectric^21^ devices, field-effect transistor (FET)^1^,^16^, thermoelectric materials^22^,^23^ and electrode^11^,^24^. Currently, increasing efforts are devoted to the construction of organic nanosheets with 2D extended *π*-conjugated systems seeking for graphene analogues with specific optical–electric properties^25^,^26^,^27^,^28^,^29^. Benefiting from the abundance of building blocks, organic 2D materials possess great diversities in physical and chemical properties as well as topological structures comparing with inorganic 2D materials, which would facilitate the applications demanding material design. Moreover, they could be prepared through wet-chemistry procedures, which offer the advantages of well-controllable tuning of the architectures and dimensionality as well as cost efficiency. Presently, organic *π*-conjugated 2D materials could be realized through two manners, one is *π–d* conjugated coordination polymers (CPs)^30^ composed of metal ions and *π*-conjugated ligands, the other is *π*-conjugated polymers with covalent-bonded aromatic units^31^. More and more results show that construction of 2D CPs is relatively accessible for achieving intralayer delocalized *π*-electron systems^30^,^32^,^33^, as 2D *π*–*d* conjugation can be rendered by suitable combination of the metal ions and chelating units that lead to the hybrid of the *d*-orbits of transition metals and frontier orbits of conjugated ligands.

As a multidentate ligand with sixfold symmetry, benzenehexathiol (BHT) has received great experimental and theoretical research interests. In an earlier work, a three-dimensional (3D) CP [Pb_3_(C_6_S_6_)]_*n*_ with an optical band gap of 1.7 eV was reported^34^, which highlighted the potential of metal–BHT systems for CPs with interesting physical properties. Lately, a semiconducting nanosheet composed of 2D layer structure of nickel–BHT complex (Ni-BHT) was prepared by Nishihara and co-workers^30^. Further spectroscopy characterization and refined electrical conductivity measurement revealed its metallic nature and oxidation-tunable conducting behaviour^33^. These reports have inspired great theoretical interests on BHT-based CPs^35^,^36^,^37^,^38^,^39^, which lead to the prediction of organic topological insulator^40^ and half metallic spin lattice^41^. Besides nickel, copper ion is another widely employed transition metal in the studies of conductive coordination compounds. From bisdithiolene complexes^42^, charge transfer salts with tetracyanoquinodimethane^43^,^44^, N,N-dicyanoquinonediimine derivatives (DCNQIs)^45^,^46^, to one-dimensional (1D) conducting CPs^47^ and 2D networks^48^, copper complexes have been demonstrated to be of great potential for the development of conducting and magnetic materials. Therefore, it is worth to explore the CPs formed by copper ion and BHT. Besides this consideration, our recent findings of the high-performance organic thermoelectric materials based on metal–tetrathioethene CPs^49^ are intriguing us to explore novel conducting CPs. Here we would like to report a novel copper bis(dithiolene) complex (Cu-BHT) with planar 2D lattice and a formula of [Cu_3_(C_6_S_6_)]_*n*_. Thin films formed by the coordination of copper(II) ion and BHT at the organic/aqueous interface were found to be composed of the nanosheets of this Cu-BHT complex. These thin films display highly electrical conducting behaviour from room temperature down to 2 K with conductivity up to 1,580 S cm^−1^ under room temperature, which is the highest value ever reported for CPs. The charge carrier transport behaviour is rationalized by band structure calculation based on the lattice structure derived from X-ray diffractions.

## Results

### Synthesis of Cu-BHT films

**Figure 1:**
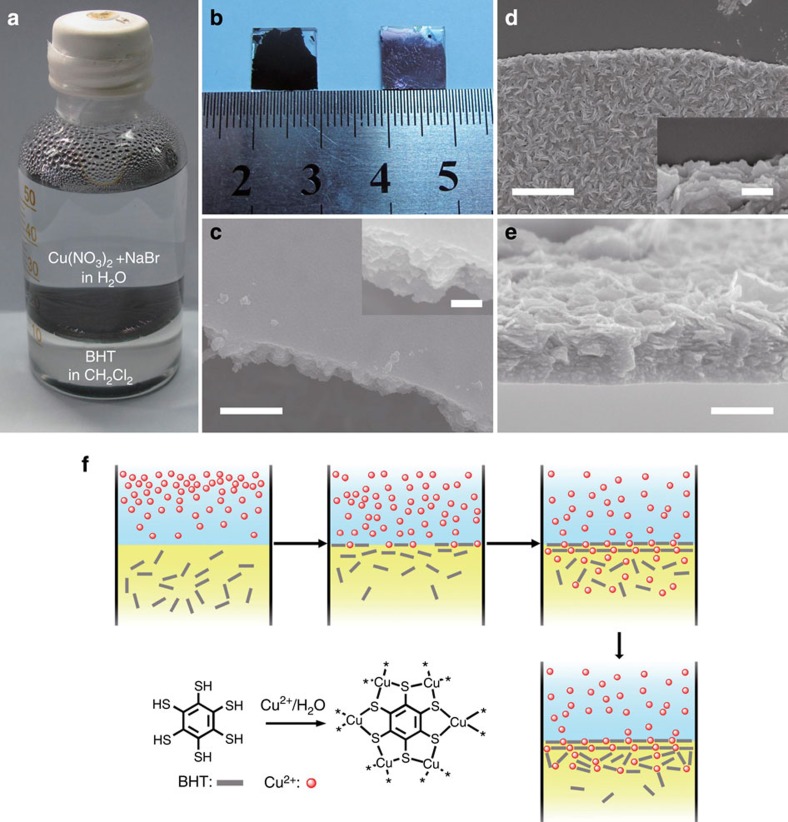
The formation of Cu-BHT films. (**a**) Photograph of the Cu-BHT film forming at the interface between aqueous solution and CH_2_Cl_2_. (**b**) Photograph of upside-up (right) and upside-down (left) films transferred on glass substrates. The SEM image of (**c**) upside, (**d**) downside surface and (**e**) cross-section of a 200-nm-thick film, the inset in **c**,**d** show detail of the film edge with enlarged scale. Scare bar, 200 nm in **c**, 400 nm in **d**,**e** and 100 nm in inset. (**f**) Scheme of the formation of Cu-BHT film.

Thin films of Cu-BHT were prepared via an interface reaction similar to that of the preparation of the Ni-BHT complex^30^. Formation of the films at the interface between aqueous and organic phases can be observed with naked eyes (Fig. 1a). The initially formed thin film was nearly transparent and turned opaque on getting thicker. The as-prepared film was then transferred on the substrate and cleaned with ethanol and acetone. Two transfer methods (detailed description can be found in the Methods section) were employed to obtain upside (the surface contacting with the aqueous phase)-up film and upside-down one, respectively. By implementing such procedures, we can get continuous films larger than 1 cm^2^ (Fig. 1b). It can be seen from the optical image that the upside surface of film is smooth and shining with metallic lustre, while the downside is rougher and matte black.

The scanning electron microscope (SEM) images show more detailed morphology difference of the two sides of the films. The upside (Fig. 1c) was flat with small roughness and quite continuous in a large scale without any steps or cracks; the other side (Fig. 1d) is much rougher because of the random orientated plate-like nanosheets. At the edges of these films, layered structures could be observed (inset in Fig. 1c,d). The cross-section SEM image of a 200-nm-thick film is shown in Fig. 1e, which indicates that the film contains plate-like sheets with thickness of several nanometres. In the area close to the upside surface, these nanosheets are closely packed parallel to the film surface; on the other side, these nanosheets stack looser with relatively random orientations. The atomic force microscopy (AFM) images of films with different thickness (ranging from 20 to 140 nm) show that the roughness of the downside surface becomes larger with the increase in film thickness (Supplementary Fig. 1), while the morphologies of the upside keep unchanged. It implies that the growth of Cu-BHT films happened at the surface of initially formed thin film facing the organic phase (the downside surface), as BHT captured copper ions and carried them from the aqueous/organic interface to the organic phase, finally crystallized there. At the beginning, plate-like sheets formed and were orderly packed parallel to water/CH_2_Cl_2_ interface because of the interface-confining effect; thus, the initially formed films were flat^50^. When the film grew thicker, the interface-confining effect became weaker. Therefore, the later-formed nanosheets became less ordered, which made the film surface rougher (Fig. 1f). To minimize the possible negative impact of this phenomenon on the electrical properties of Cu-BHT films, the reaction time should be carefully controlled to get films with appropriate thickness.

### Structural resolution

**Figure 2:**
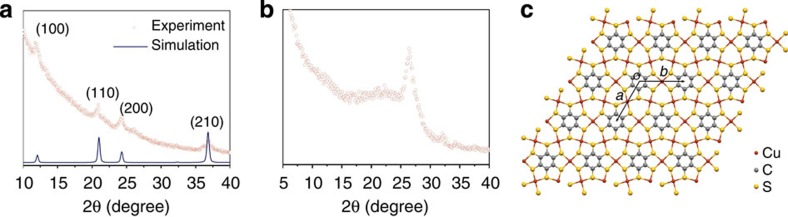
Structural characterization of Cu-BHT films. (**a**) GIXRD pattern of Cu-BHT thin film obtained with synchrotron radiation (*λ*=1.5398 Å): in-plane diffraction and a simulated pattern with a hexagonal lattice of *a*=*b*=8.45 Å and (**b**) out-of-plane diffraction. (**c**) Two-dimensional lattice of [Cu_3_(C_6_S_6_)]_*n*_ derived from the in-plane GIXRD pattern (*a*=*b*=8.76 Å).

For structure characterizations of the Cu-BHT thin film, grazing incident X-ray diffractions (GIXRD) were performed with synchrotron radiation. The in-plane and out-of-plane diffraction patterns of the same film with thickness of 60 nm were recorded separately (Fig. 2a,b). The out-of-plane diffraction pattern displays a primary peak at 26.3±0.3 ° corresponding to the interlayer distance of 3.38±0.03 Å. This indicates the formation of the layered structure parallel to the substrate. The broad diffraction peak indicates small crystalline domain of this thin film. Average size of 3 nm could be calculated from the full-width at half-maximum of the out-of-plane peak according to the Scherrer's equation, which refers to the thickness of the crystalline domains along *c* axis. In the in-plane diffraction pattern, a serials of peaks could be indexed to (*hk*0) of a hexagonal lattice with *a*=*b*=8.45 Å. A domain size of 16 nm could also be calculated. It means that the film was composed of highly oriented crystallized nanosheets. This is consistent with what observed in SEM side view of the thin films (Fig. 1e). For films with thickness exceeding 200 nm, other peaks besides the (00*l*) series could be observed in the out-of-plane patterns, which indicate the existence of random oriented nanosheets (Supplementary Fig. 2).

**Figure 3:**
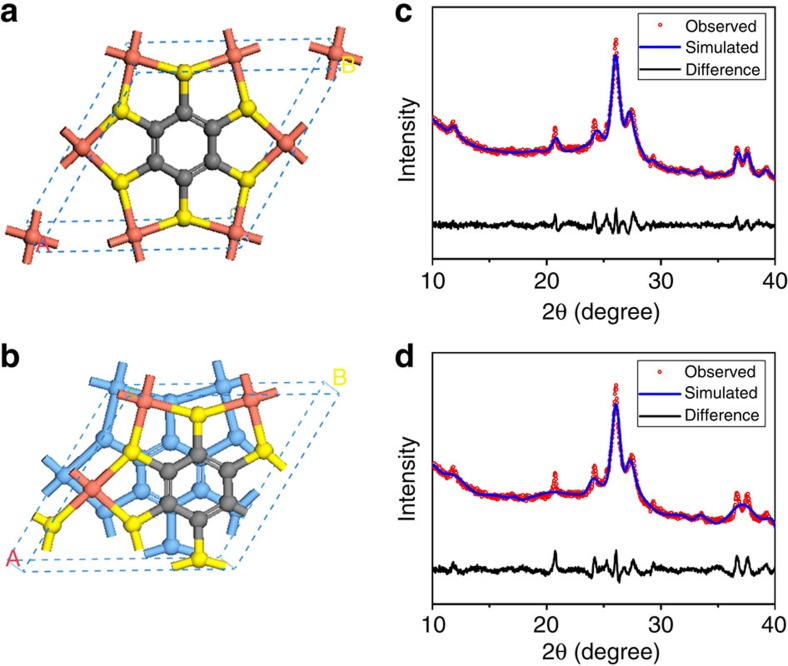
The crystal structure simulation of Cu-BHT. (**a**) AA stacking and (**b**) slipped AB stacking pattern. (**c**,**d**) Corresponding PXRD of (Cu_3_C_6_S_6_)_*n*_ thin film (red dot) and Pawley-refined XRD pattern (blue line).

On the basis of the structure parameters obtained from GIXRD, a 2D lattice was proposed as [Cu_3_C_6_S_6_]_*n*_ (more discussion can be found in Supplementary Note 1). As show in Fig. 2c, one BHT connects with other six BHTs through the shared Cu atoms, forming a lattice with sixfold symmetry. Each Cu atom coordinates with four S atoms in a square-planar manner, resulting in a dense topological structure without obvious pores. If the six-membered carbon rings of phenyl moieties are ignored, a continuous 2D Cu–S network could be observed. This 2D lattice was further optimized by DFT-PBE (density functional theory Perdew–Burke–Ernzerhof) calculation that results in a hexagonal cell with *a*=*b*=8.76 Å that is in good agreement with the experiment data (*a*=*b*=8.45 Å). To further clarify the interlayer stacking pattern, we made potential energy surface (PES) scan fixing an interlayer separation of 3.38 Å according to GIXRD data; single-point energy calculations were carried out using the General Gradient Approximation–PBE exchange correlation functional with D2 dispersion correction for structures with ∼1,000 different *ab* plane displacements. The PES for AA and AB stacking pattern is shown in Supplementary Fig. 3 and corresponding crystal structures with the lowest energy are shown in Fig. 3. Since the minor energy difference (∼65 meV) for AA and AB stacking pattern, there probably exists a mixture of AA and AB stacking configuration. Experimental powder X-ray diffraction (PXRD) was recorded on finely grounded thin films with an X-ray diffraction meter using Cu Kα irradiation (*λ*=1.5406 Å). On the basis of our optimized crystal structure, the PXRD data could be reproduced by Pawley refinement without modifying crystal lattice and atoms' position. The tiny difference between the observed and simulated PXRD (See Fig. 3c,d) supported our proposed crystal structure and detailed crystal structure parameters can be found in Supplementary Table 1.

### Component analysis

The electron probe microanalysis results of Cu-BHT films indicate the existence of copper, oxygen, sulfur and carbon (Supplementary Fig.4), and the calculated atom ratio of copper to sulfur is ∼0.54, which is close to 1:2 (see Supplementary Table 2). Using elemental analysis (EA), although the exact amount of sulfur cannot be determined because of the presence of copper, the weight percentage of carbon was determined to be 16.4%, which is coincident with the calculated result (15.8%) of the formula of [Cu_3_C_6_S_6_]_*n*_. The full spectrum of X-ray photon spectrum (XPS) of Cu-BHT film is obtained (Supplementary Fig. 5) that verifies the absence of sodium, chlorine, nitrogen and bromine, which were contained in the starting reagents. Combining the results mentioned above, the formula of [Cu_3_C_6_S_6_]_*n*_ is quite reasonable.

### Electrical conductivity

**Figure 4:**
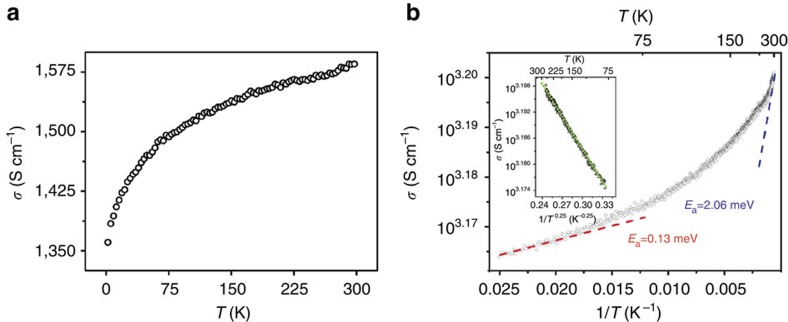
Temperature dependence of electrical conductivity of a Cu-BHT film. (**a**) Electrical conductivity (*σ*) of a 150-nm film as a function of temperature ranging from 2 to 300 K. (**b**) Plots of *σ* (*T*) versus *T*^−1^ and (inset in **b**) *σ* (*T*) versus *T*^−0.25^. This measurement was performed on a film with the thickness of 400 nm by four-probe method. *I–V* curve have been recorded to make sure the measurement was performed in the Ohmic region (see Supplementary Fig. 6).

The electrical conductivities of Cu-BHT films with different thickness (15–500 nm) were measured using a standard four-probe method. It was found that all of the films measured possess high electrical conductivities ranging from 750 to 1,580 S cm^−1^, which are irrelevant with the thickness of the films. The highest value is higher than that of conductive CPs ever reported, and even surpasses Cu-DCNQI single crystal^51^ and 1D [Pt_2_(*dta*)_4_I]_*n*_ (*dta*=dithioacetato) defect-free nanoribbons^52^. Meanwhile, a weak temperature dependence of conductivity was observed. As shown in Fig. 4a, a film sample (thickness of 400 nm) with room temperature conductivity of 1,580 S cm^−1^ maintains high conductivity (1,360 S cm^−1^) at 2 K. The ratio of *σ*(300 K)/*σ*(2 K) is equal to 1.16. Similar behaviours have been observed on the highly conducting polymers with ordered structure, such as PEDOT:OTf^53^. Moreover, this sample shows a nonlinear relationship between the logarithm of the conductivity and the reciprocal of the absolute temperature (Fig. 4b), which is commonly observed in conducting polymers or CPs^54^. The slope of the curve can be seen as *E*_a_/*k*, where *E*_a_ is the activation energy of the electrical conductivity and *k* is the Boltzmann constant. The *E*_a_ continuously rises with increasing temperature and ranges from 0.12 meV at 40 K to 2.06 meV at 300 K, which is extremely small especially in low temperature range. As shown in the inset of Fig. 4b, the plot of log_10_ *σ* versus *T*^−0.25^ over the temperature region 40–250 K are well fitted to the 3D variable range hopping model. It implies that the hopping process between nanosheets should be the critical step for electrical conducting process of the Cu-BHT film. This is coincident with what revealed by GIXRD and SEM characterizations that the polycrystalline films are composed of densely stacked nanosheets, and defects and boundaries are existing between adjacent crystalline domains. To get more information about the conducting mechanism of this 2D CPs, thermopower measurements were carried out under room temperature, as it is much less susceptible to the resistive boundaries for a zero-electrical-current measurement. The thermopower of Cu-BHT films ranges from −4 to −10 μV K^−1^. Such small values are typically observed in metals or highly conducting polymers with metallic behaviour^53^, which suggests the metallic nature of Cu-BHT films.

**Figure 5:**
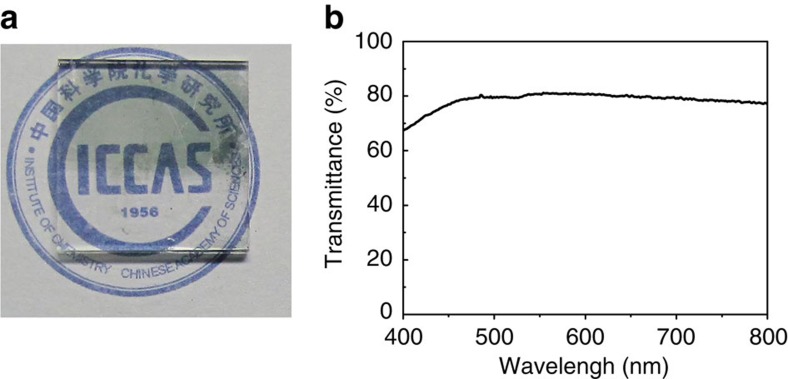
Optical properties of the Cu-BHT film. The photograph (**a**) and UV-Vis spectrum (**b**) of the Cu-BHT film. In order to make a reference, lower half of the film on glass substrate (1.1 × 1.1 cm^2^) have been removed. The thickness of this film is 60 nm. The average transmittance is calculated to be 78.6%.

The ultrathin Cu-BHT films looked semitransparent, which encouraged us to evaluate its possibility to serve as transparent electrode. The photograph and ultraviolet-Vis absorption spectrum of a 60-nm-thick film are shown in Fig. 5. The film has no obvious absorption in the visible region, and the average transmittance is up to 78.6%. By performing a four-point probe measurement, a low sheet resistance value of 200 Ohm per square (Ω □^−1^) was observed, which highlights its potential to be a new choice for transparent electrodes. The most attractive advantage for this material is that it could be fabricated through a low-cost solution process.

### Characterization of Cu-BHT-based FET

**Figure 6:**
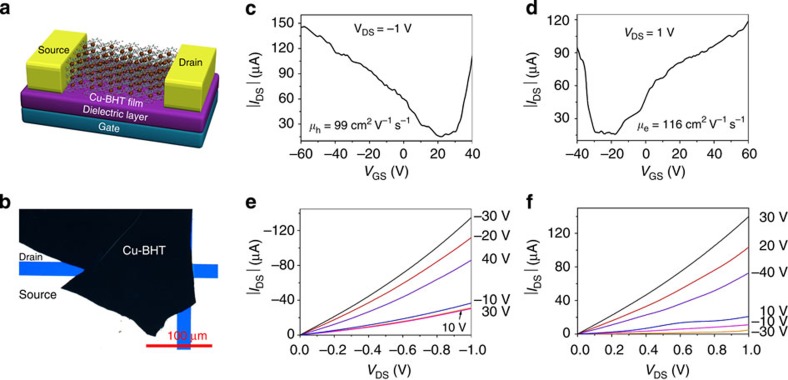
Fabrication and characterization of Cu-BHT FET. (**a**) Illustrative schematic of Cu-BHT-based field-effect transistors. (**b**) Photograph of bottom-gate bottom-contact FETs based on the Cu-BHT film. (**c,d**) Output and (**e,f**) transfer characteristics of Cu-BHT-based FETs.

To further characterize the charge transport properties of this 2D CPs, FETs based on Cu-BHT films were fabricated and characterized with bottom-gate bottom-contact device geometry (Fig. 6a,b). As mentioned above, two sides of the films have different surface morphology. It is necessary to make the smooth upside surface contacting with the insulated layer and electrodes to get the devices work. The output and transfer curves of a typical device are shown in Fig. 6c–f, which display ambipolar behaviour. The field-effect mobility is extracted from *I*_DS_–*V*_GS_ curves in the linear regime as described in the experimental section. Both high hole and electron mobility over 10 cm^2 ^V^−1 ^s^−1^ can be routinely obtained. Notably, a prominent hole mobility of 99 cm^2 ^V^−1 ^s^−1^ and electron mobility of 116 cm^2 ^V^−1 ^s^−1^ was achieved in a same device, indicating excellent and balanced ambipolar charge transport properties of Cu-BHT. The results represent the first successful modulation of charge transport properties in FET geometry with CP and the performance surpasses all the reported mobilities of organic ambipolar devices. At the same time, these devices show *I*_on_/*I*_off_ ratio of 10. The coexistence of the relative low on/off ratio and high mobility is common for the typical gapless graphene-based FETs^55^. The on/off ratio in graphene-based FET is tunable by using graphene nanoribbons as a channel to open a band gap or using two graphene-layer structure. There are different ways to manipulate the electronic band structure of CPs such as doping^56^ and oxidation/redox control^33^, which highlight its potential application in the FET device.

### XPS and UPS characterizations

**Figure 7:**
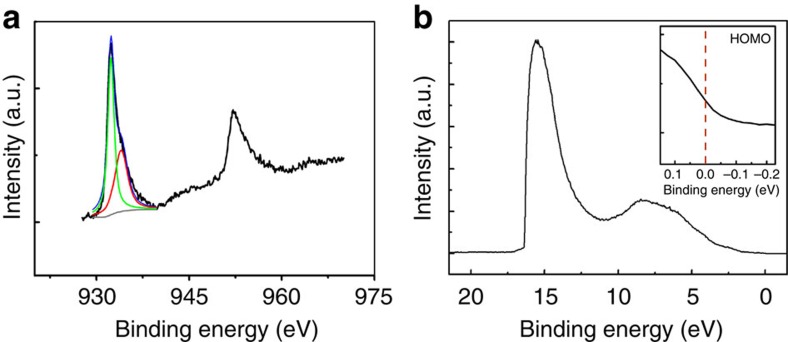
XPS and UPS characterizations of the Cu-BHT film. (**a**) XPS focusing on the Cu 2*p* region and (**b**) UPS of Cu-BHT film acquired at 300 K. The inset is detailed UPS of the Fermi edge.

To understand the origin of the highly conducting property of this coordination network, XPS and ultraviolet photoemission valance band spectrum (UPS) of the film were observed at 300 K (Fig. 7), the XPS was focused on the Cu 2*p* regions. The binding energy of 953.1 and 932.3 eV correspond to the Cu 2p 1/3 and 2p 2/3 receptivity. The lack of shake-up structure suggests the absence of Cu(II). The Cu 2p 2/3 peak near 932.6 eV matches well with the binding energy value of a typical Cu 2p 2/3 peak of Cu(I) compound, which means the Cu(II) ion have been reduced during the formation of Cu-BHT^57^. The peaks of both Cu 2*p* are asymmetric, which suggests that there should be two kinds of Cu with different chemistry environment. Therefore, the Cu 2p 2/3 peak is discerned into 932.6 and 934.1 eV. The wide peak at 934.1 eV with smaller intensity may belong to Cu atom at the surface layer or edge of the nanosheets. The charge transfer between ligands and metal ions implies strong *π–d* interaction and heavier electron delocalization among the 2D lattice that contribute to its highly conductive behaviour. Figure 7b shows the UPS of the film. The observation of Fermi edge (inset in Fig. 7b) further confirms the metallic nature of Cu-BHT thin film. On the other hand, electronic structure calculations have been performed for AA and AB stacking crystal on the basis of PBE (Fig. 3), and many-body perturbation correction *G*_0_*W*_0_ approach (Supplementary Fig. 7). Irrespective of AA or AB stacking and single-layer configuration (Supplementary Fig.8), the Fermi level cuts the very wide bands that gives rise to a large carrier concentration along with high mobility and thus reveals metallic behaviour and high conductivity.

## Discussion

In summary, the Cu-BHT thin films composed of 2D nanosheets of [Cu_3_(C_6_S_6_)]_*n*_ were synthesized and characterized. By using an easy accessible interfacial reaction and proper transfer method, large-scale thin films composed of highly oriented nanosheets have been prepared. A convincing structure was derived from PXRD and GIXRD analyses and verified by quantum simulation. High electrical conductivity of up to 1,580 S cm^−1^ at 300 K was observed, which is the highest value ever reported for CPs. It also displays good transmittance in the visible region and small sheet resistances, which highlights its potential to serve as transparent electrodes. Furthermore, the electrical conductivity of the Cu-BHT films can be modulated by electric field with high mobilities of electron (116 cm^2 ^V^−1 ^s^−1^) and hole (99 cm^2 ^V^−1 ^s^−1^) at 300 K. UPS and first-principles calculations confirmed the metallic nature of this 2D CPs. It should be noticed that even the thinnest films synthesized at the present stage contain dozens of layers of [Cu_3_(C_6_S_6_)]_*n*_ 2D lattice. Owing to the multicrystalline nature and relatively small domain size of these thin films, the transport behaviours presented here is representative of a bulk material, while the impacts of defects and domain boundaries still could not be ruled out. For investigating the intrinsic transport properties of the [Cu_3_(C_6_S_6_)]_*n*_ lattice, preparation of high-quality single crystals and large-size monolayers are highly desirable. Currently, alternative preparing methods such as Langmuir–Blodgett and chemical vapour deposition methods are under investigation for achieving precise control of the film thickness and enlarging the crystalline domain size.

## Methods

### Materials

Cu(NO_3_)_2_ and NaBr were purchased from Alfa Aesar China (Tianjin) Co., Ltd. Dichloromethane was purchased from Arcos Organics Co. Water was purified using the Milli-Q purification system. Both solvents were degassed with the freeze–thaw method before using. BHT was synthesized according to the literatures^58^.

### Synthesis of Cu-BHT thin film

The thin film of Cu-BHT was prepared via a reaction between Cu(II) nitrate and BHT at the interface of dichloromethane–water. Under argon atmosphere, BHT was first dissolved in degassed dichloromethane to afford a saturated solution (0.24 mM). The solution (20 ml) of BHT was added to a sealed bottle filled with argon gas and then covered with degassed water (20 ml) to form an oil–water interface. The aqueous mixture of Cu(NO_3_)_2_ (5 mM) and NaBr (1 mM) was then added into the water gently and slowly (0.5 ml min^−1^) by using a syringe pump. Formation of the film can be observed with naked eyes at the dichloromethane–water interface.

### Transfer method

For spectroscopy, structure and electrical property characterizations, quartz glasses, glass slides and silicon wafers covered with 300 nm of SiO_2_ were used as substrates, respectively. Two transfer manners similar to that of Langmuir–Schaefer transfer method were employed for getting upside-up and upside-down films. To get the upside-up film, a substrate was settled on a holder horizontally in the reaction container before adding the reaction solutions, and then a solution of BHT in CH_2_Cl_2_ was filled in to make the surface of the solution ∼10 mm higher than the substrate. After the Cu-BHT thin film formed, the subphase was removed from the container with a syringe to make the film deposit on the substrate with the CH_2_Cl_2_/aqueous interface passing through the substrate. The upside-down films were obtained by immerging the substrate into the reaction solution and passing though the CH_2_Cl_2_/aqueous interface horizontally and slowly, after the Cu-BHT film formed. Then, the aqueous solution was removed before the substrate was taken out of the CH_2_Cl_2_ solution.

### SEM and AFM characterizations

SEM images of the Cu-BHT films were taken with a Hitachi S4800-SEM with acceleration voltage of 5 K volt. Before being tested, the films were transferred to conductive silicon substrates and then covered with platinum thin films with a thickness of several nanometres. AFM images were obtained by using a Digital Instrument NanoScope IIIa AFM with tapping mode. The roughness was calculated from the AFM images by using the NanoScope Analysis software.

### Component characterization and analysis

Entire element analysis of the film was performed with electron probe microanalysis (JEOL, JXA-8,100). The content of carbon was analysed using Flash EA 1112 (Thermo Fisher Scientific).

### X-ray characterization and analysis

Out-of-plane and in-plane diffraction patterns of the Cu-BHT thin films were obtained at diffuse X-ray scattering beam line of Beijing Synchrotron Radiation Facility (*λ*=1.5398 Å), from 2*θ*=5 to 40° with 0.05° increment at room temperature. PXRD of grounded Cu-BHT thin films were recorded on *D*/max 2,500 with Cu Ka source (*λ*=1.5406 Å). Pawley refinement was carried out with crystal structures with the lowest energy obtained in PES search. Reflex, a software package for crystal determination from XRD pattern, implemented in MS modelling Ver 7.0 (Accelrys Inc.) was employed. Peak-broadening (pseudo-Voigt function), asymmetry correction (Berrar–Baldinozzi function) and zero-shift errors were refined together to achieve the improved profile fitting. The refinement converged to Rwp=4.98%, Rp=3.78% and Rwp=6.54%, Rp=4.70% for the AA and AB stacking patterns, respectively.

### Electrical property measurement

To measure electrical property of Cu-BHT thin films, they were transferred on the insulated substrate (quartz) and then four parallel gold electrodes were deposited on them. The electrical conductivity was measured by a four-electrode set-up in a Physical Property Measurement Systems (PPMS-9, Quantum design) with a constant current of 1 μA. The temperature dependence of conductivity was tested in the sealed sample chamber of PPMS, which can sweep the temperature from 300 K to 2 K, with deviation less than 0.01 K. The voltage drop between two electrodes inside can be recorded online along with the change of temperature. The resistance was calculated from the voltage drop divided by constant current, which was converted to conductivity by calculating with width/length ratio and thickness, which was determined using AFM.

The sheet resistance of 60-nm-thick Cu-BHT thin film was measured in a ST2263 Double Testing Digital Four-probe Tester (Suzhou Jingge Electronic Co. Ltd.) with a constant current of 100 μA at room temperature. Before measurement, the film was deposited on an 11 × 11 mm^2^ glass substrate. Because the sample's resistivity is low, the configuration of four-point probes in-line was performed with probe spacing of 1 mm, and force of 3 N was applied to the probes to make sure of good contact between probes and sample.

### Measurement of thermopower

Two parallel gold electrodes (with a thickness of 30 nm) with a segregation of 5 mm were deposited on a Cu-BHT thin film that had been transferred on a SiO_2_ substrate. Then, the SiO_2_ slide settled with one side on a Peltier cooler and the other side on a Peltier heater, which were employed to apply a temperature gradient across the gap between the gold electrodes. The temperature difference and thermoelectric potential between the gold electrodes were determined with a Thermal Imaging Cameras (FLIR A300, FLIR Systems Inc.) and a Keithley 4200 SCS semiconductor characterization system (Keithley Instruments Inc.), respectively. Then, the thermopower was calculated by dividing the thermoelectric potential by temperature difference.

### Light absorption spectrum

Light absorption spectrum of the same film used in sheet resistance measurement was recorded on a CIMPS system (ZAHNER) for photoelectrochemical transmittance/absorbance consisting of balance Tungsten–Deuterium as the light source (wavelength from 220 to 1,100 nm), two optical fibres for transmission of the light, STE-BLK-CXR-SR Concave Grating Spectrometer for collecting the light signal passed through the sample and the CIMPS-abs software for analysing the signal. The blank glass substrate was used for reference.

### Fabrication and characterization of FETs

The Cu-BHT thin film was deposited on the n-type highly doped Si substrate with 300 nm SiO_2_. The source and drain electrodes (30 nm) were sputtered and patterned using the lift-off technique. The channel length is 5∼50 μm. Before deposition of Cu-BHT, the substrates were rinsed with deionized water, ethanol and acetone. The Cu-BHT film was rinsed with deionized water and methylene chloride to remove the residual impurities after it was transferred on the substrate. The obtained films were dried overnight in the glove box with H_2_O concentration <0.1 p.p.m.

The device characteristics were measured at room temperature with a Keithley 4,200 SCS semiconductor parameter analyser. The mobility was calculated in the linear regime form the equation *I*_DS_=(*WμC*_i_/*L*)(*V*_GS_−*V*_T_)*V*_DS_, where *I*_DS_ is the drain-source current, *μ* is the filed-effect mobility, *W* and *L* are the channel width and length, respectively, *C*_i_ is the capacitance per unit of the dielectric layer, *V*_T_ is the threshold voltage, and *V*_GS_ and *V*_DS_ are the gate voltage and drain-source voltage, respectively.

### XPS and UPS characterization

XPS and UPS were characterized by using AXIS Ultra-DLD ultrahigh vaccum photoemission spectroscopy system (Kratos Co.). A monochromatic aluminium K_α_ source (1,486.6 eV) and a He I source (21.11 eV) were used for XPS and UPS, respectively. All the characterizations were performed in a base pressure of better than 3 × 10^−9 ^Torr. Negative substrate bias voltage of 9 volt was implemented during the UPS measurement. Before measurements, the Cu-BHT films were transferred on highly doping conductive silicon substrates (1.0 × 1.0 cm^2^).

### Calculation detail

The first-principle calculations are performed by the projector-augmented wave method as implemented in the VASP package^59^. Energy scan and single-point calculation were performed with a 5 × 5 × 11 Monkhorst-Pack K-mesh and PBE functional for AA stacking (and 5 × 5 × 5 K-point for AB configuration). On the basis of previous self-consistent charge density, the band structure at the PBE level of AA and AB stacking patterns can be obtained, as shown in Supplementary Fig. 8. In addition, on top of PBE wave function, many-body perturbation correction *G*_0_*W*_0_ approach has been performed. Plane waves have been included up to an energetic cutoff of 600 eV. Gamma centred 2 × 2 × 4 *k*-mesh integration within the Brillouin zone and 240 empty bands have been chosen. Through Wannier interpolation method^60^, the band structure have been obtained for AA stacking configuration.

## 

## Additional information

**How to cite this article:** Huang, X. *et al.* A two-dimensional *π–d* conjugated coordination polymer with extremely high electrical conductivity and ambipolar transport behaviour. *Nat. Commun.* 6:7408 doi: 10.1038/ncomms8408 (2015).

## Supplementary Material

Supplementary Information Supplementary Figures 1-9, Supplementary Tables 1-3, Supplementary Note 1 and Supplementary References
