# Supplementary material for: A two-dimensional π–d conjugated coordination polymer with extremely high electrical conductivity and ambipolar transport behaviour
Source: Nat Commun. 2015 Jun 15;6:7408. doi: 10.1038/ncomms8408 (PMC4490364; doi:10.1038/ncomms8408)
Supplement: Supplementary Information — Supplementary Figures 1-9, Supplementary Tables 1-3, Supplementary Note 1 and Supplementary References [file ncomms8408-s1.pdf]

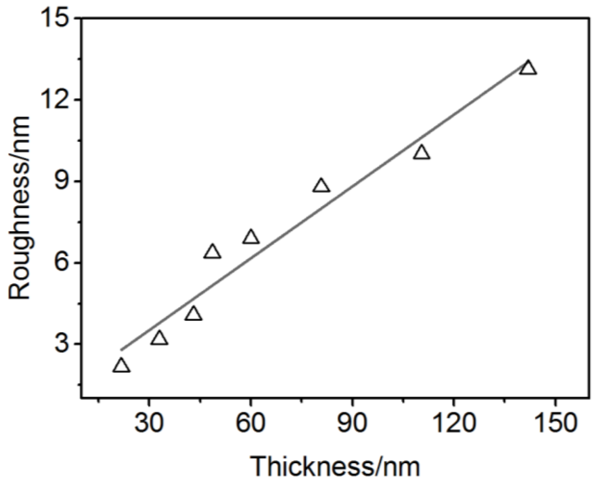

Supplementary Figure 1. Roughness of **Cu-BHT** films with different thickness.

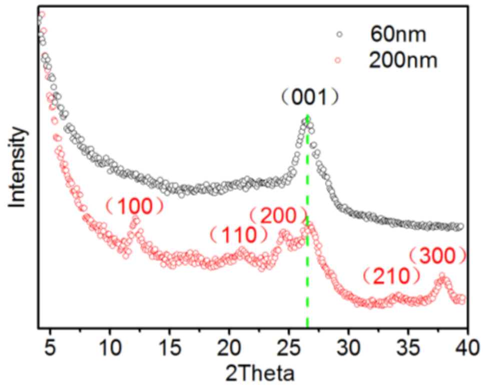

Supplementary Figure 2. GIXRD out-plane pattern different between thin film of 60 nm and thicker film of 200nm

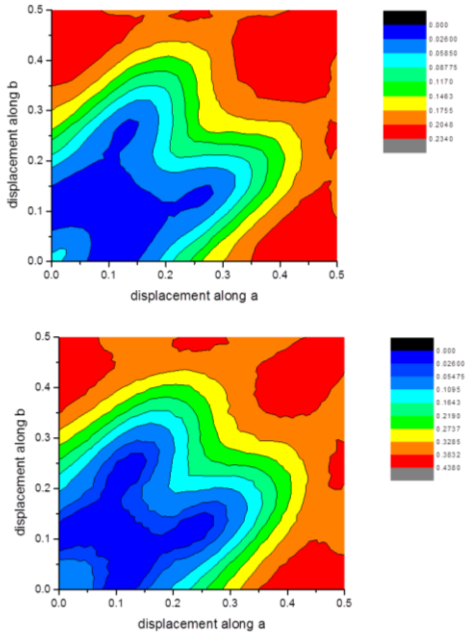

Supplementary Figure 3. The potential energy surface (PES) for AA and AB stacking pattern along a, b lattice axis, the total energy at the origin point is shifted to zero.

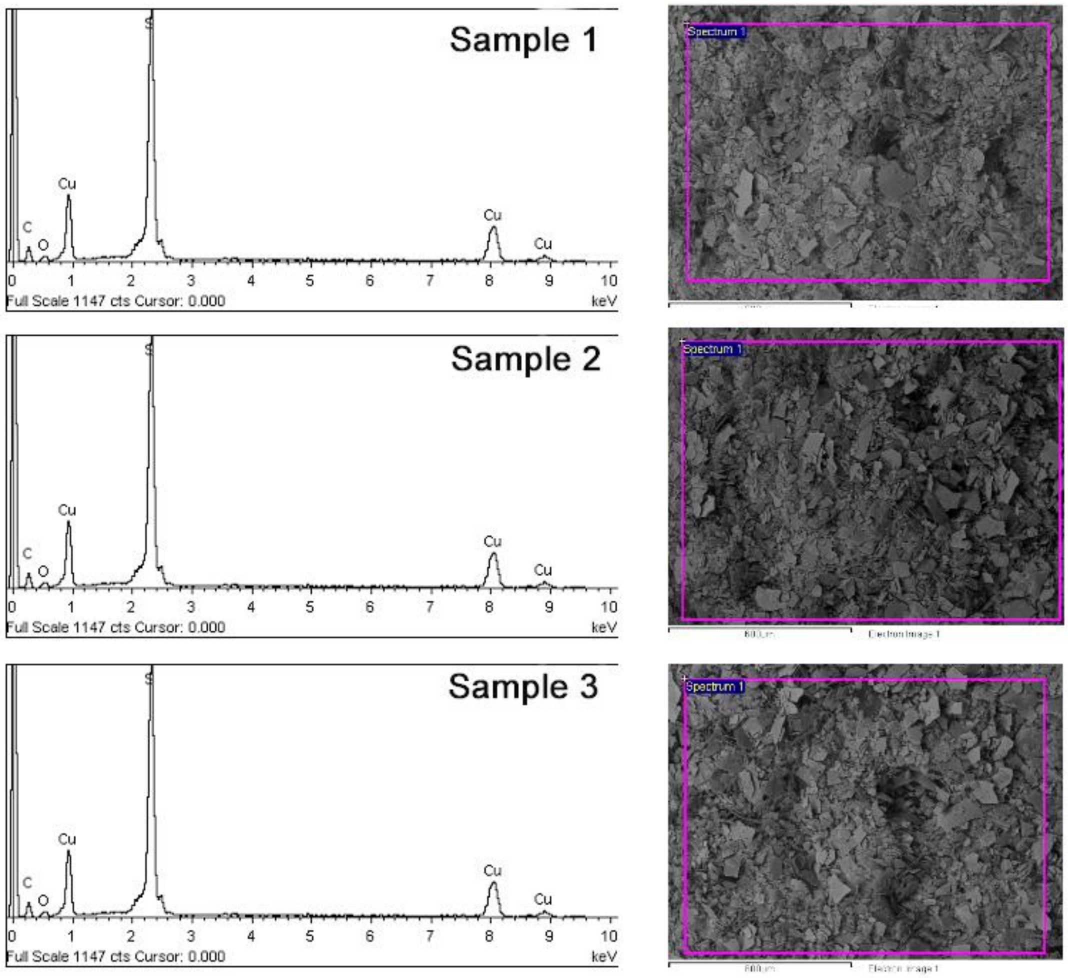

Supplementary Figure 4. EPMA spectrum of (left) corresponding to each area of **Cu-BHT** samples in SEM image (right)

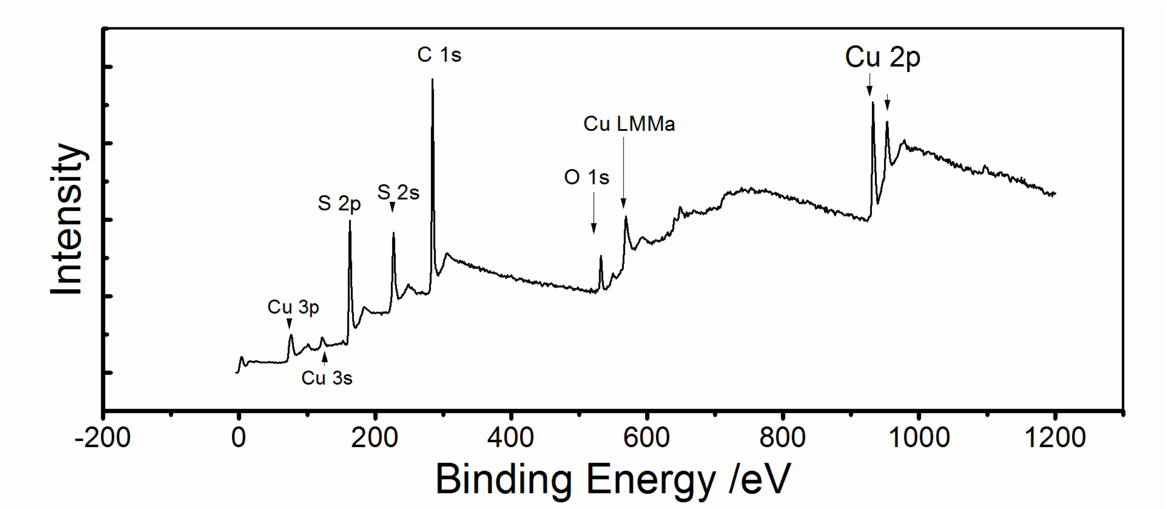

Supplementary Figure 5. XPS full spectrum of **Cu-BHT** film

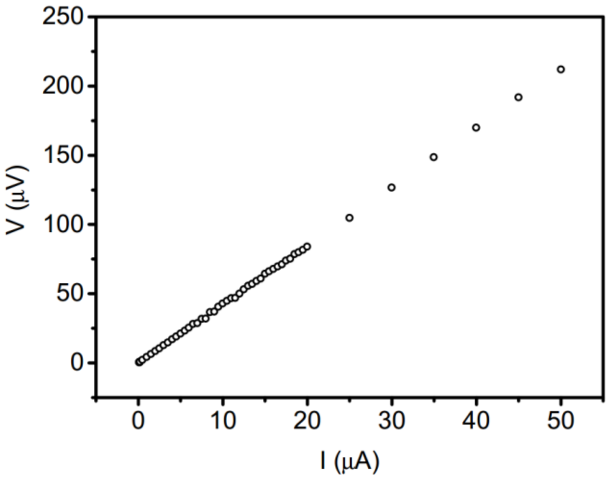

Supplementary Figure 6. IV-Curve of **Cu-BHT** film

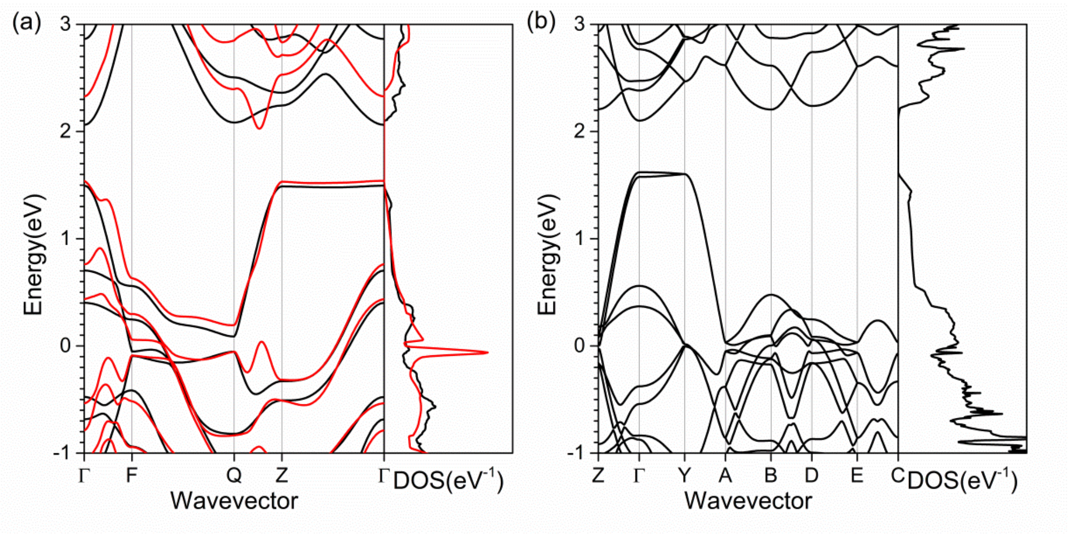

Supplementary Figure 7 (**a**) Band structure and total density of state (DOS) for AA stacking pattern based on PBE level (black line) and G_0W_0 level (red line). High-symmetry K points: Γ=(0,0,0), F=(0, 0.5,0), Q=(0,0.5,0.5), Z=(0,0,0.5); (**b**) Band structure at PBE level for AB stacking pattern. High-symmetry K points: Z=(0.5,0,0), Γ=(0,0,0), Y=(0,0,0.5), A=(0,-0.5,0.5), B=(0,-0.5,0), D=(0.5,-0.5,0), E=(0.5,-0.5,0.5), C=(0.5,0,0.5). The Fermi level is at zero.

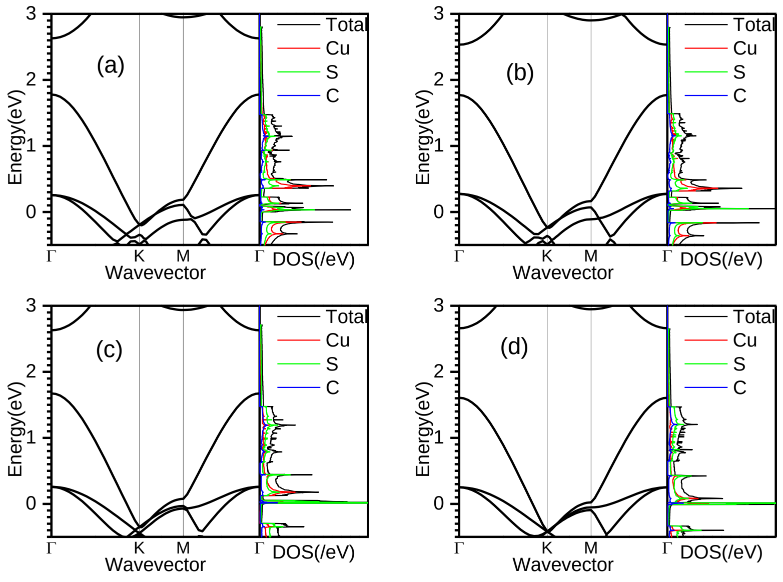

Supplementary Figure 8. Band structure and Density Of State (DOS) at (a)PBE, (b)LDA, (c) LDA+U(U=4), and (d) LDA+U(U=6) level for single layer. K point Γ=(0,0,0), F=(0,0.5,0), Q=(0,0.5,0.5), Z=(0,0,0.5), X=(0.5,0,0), A=(0,-0.5,0.5), F'=(0,-0.5,0), D=(0.5,-0.5,0), E=(0.5,-0.5,0.5), C=(0.5,0,0.5). The Fermi level is at zero.

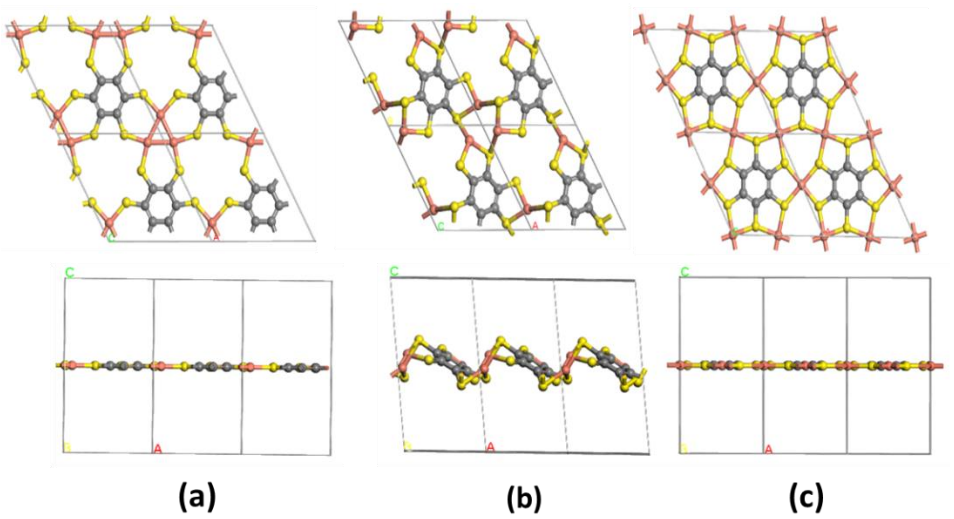

Supplementary Figure 9. Theoretical predicted single layer topological structure (a), (b) and (c), top view (upper panel) and side view (bottom panel).

Supplementary Table 1. Optimized crystal structure based on the minimum of potential energy surface for AA and AB stacking with PBE-D2 functional.

Staking Pattern	a	b	c	α	β	γ
AA	8.748	8.748	3.539	77°	89.2°	120°
AB	8.748	8.748	6.82A	90°	90°	120°

Supplementary Table 2. Cu/S ratio calculated from EPMA.

	Cu/S atom ratio
Sample1	0.5481
Sample2	0.5479
Sample3	0.5383

Supplementary Table 3.The Lattice parameters for crystal (a), (b) and (c) in Supplementary Figure 9.

Lattice parameters	*a* (Å)	*b* (Å)	*c* (Å)	*α* (°)	*β* (°)	*γ* (°)	*Space group*	*Total Energy/eV*
Crystal (a)	9.39	9.39	14.9	90.00	90.00	120.00	P-6m2	-85.52
Crystal (b)	8.33	8.80	15.0	87.72	96.07	119.65	P1	-88.13
Crystal (c)	8.75	8.75	15.0	90.00	90.00	120.00	P6/MMM	-90.05

Supplementary Note 1

Considering the EPMA data and mild reaction conditions, reaction product can be inferred with formula $Cu_3C_6S_6$. Indeed, we have employed Particle-swarm optimization (CALYPSO[1]) algorithm to predict possible crystal structure with specific chemical composition. This method has been widely used in highly stable 2D nanostructures prediction[2-5]. Through thousands of possible structures screening and then structural optimization within the framework of density functional theory as implemented in the Vienna Ab initio Simulation Package (VASP)[6], several possible topological structures have been proposed, as can be seen in Supplementary Fig. 9 and corresponding lattice parameters can be found in Supplementary Table 3. It is obvious that Crystal (c) display the smallest total energy and then it is the most stable one. In Addition, its lattice parameter and symmetry are much consistent with GIXRD data (as stated in manuscript). Therefore, we have reasons to believe that Crystal (c) is the most likely topological structure.

Supplementary References

1 Wang, Y., Lv, J., Zhu, L. & Ma, Y. CALYPSO: A method for crystal structure prediction. *Comput. Phys. Commun.* **183**, 2063-2070, (2012).

2 Luo, X. *et al.* Predicting Two-Dimensional Boron–Carbon Compounds by the Global Optimization Method. *J. Am. Chem. Soc.* **133**, 16285-16290, (2011).

3 Li, Y., Liao, Y. & Chen, Z. Be_2C Monolayer with Quasi-Planar Hexacoordinate Carbons: A Global Minimum Structure. *Angew. Chem. Int. Ed.* **53**, 7248-7252, (2014).

4 Zhou, L.-J., Zhang, Y.-F. & Wu, L.-M. SiC_2 Siligraphene and Nanotubes: Novel Donor Materials in Excitonic Solar Cells. *Nano Letters* **13**, 5431-5436, (2013).

5 Wu, X. *et al.* Two-Dimensional Boron Monolayer Sheets. *ACS Nano* **6**, 7443-7453, (2012).

6 Kresse, G. & Furthmüller, J. Efficiency of ab-initio total energy calculations for metals and semiconductors using a plane-wave basis set. *Comput. Mater. Sci.* **6**, 15-50, (1996).
